# Production of Hydroxycarboxylic Acid Receptor 3 (HCA_3_) Ligands by *Bifidobacterium*

**DOI:** 10.3390/microorganisms9112397

**Published:** 2021-11-21

**Authors:** Takuma Sakurai, Ayako Horigome, Toshitaka Odamaki, Takashi Shimizu, Jin-Zhong Xiao

**Affiliations:** Next Generation Science Institute, Morinaga Milk Industry Co., Ltd., Zama 252-8583, Japan; t_sakura@morinagamilk.co.jp (T.S.); a-horigome@morinagamilk.co.jp (A.H.); t-odamak@morinagamilk.co.jp (T.O.); t_simizu@morinagamilk.co.jp (T.S.)

**Keywords:** *Bifidobacterium*, indole-3-lactic acid, 3-phenyllactic acid, 4-hydroxyphenyllactic acid, leucic acid, hydroxycarboxylic acid receptor 3, aryl hydrocarbon receptor

## Abstract

Hydroxycarboxylic acid receptor 3 (HCA_3_) was recently identified in the genomes of humans and other hominids but not in other mammals. We examined the production of HCA_3_ ligands by *Bifidobacterium* spp. In addition to 4-hydroxyphenyllactic acid, phenyllactic acid (PLA), and indole-3-lactic acid (ILA), we found that LeuA was produced by *Bifidobacterium* as an HCA_3_ ligand. The four ligands produced were the mixtures of enantiomers, and D-ILA, D-PLA, and D-LeuA showed stronger activity of the HCA_3_ ligand than their respective L-isomers. However, there was no difference in AhR activity between the two ILA enantiomers. These results provide new insights into the HCA_3_ ligands produced by *Bifidobacterium* and suggest the importance of investigating the absolute stereo structures of these metabolites.

## 1. Introduction

Hydroxycarboxylic acid receptors (HCA_1_, HCA_2_, and HCA_3_) play important roles in maintaining energy and immune homeostasis [1]. Based on their structures, they are classified as G-protein-coupled receptors [2]. Most mammals possess HCA_1_ and HCA_2_; however, only humans and other hominids have HCA_3_ in their genomes [3]. HCA_3_, also known as GPR109b, is expressed in adipocytes, macrophages, neutrophils, and colonic epithelial cells [4]. HCA_3_ is derived from the gene duplication of HCA_2_ [5], which is activated by β-hydroxybutyrate, butyrate, and nicotinic acid [6]. In contrast, HCA_3_ is activated by β-hydroxyoctanoate, which is produced by hepatocytes during the β-oxidation of fatty acids. Similar to HCA_2_, HCA_3_ inhibits lipolysis in adipocytes, leading to a negative feedback mechanism for the β-oxidation of free fatty acids [7,8]. Although HCA_2_ and HCA_3_ appear to play the same roles in the human body, some differences have been reported. Aryllactic acids (Aryl-LAs), such as 4-hydroxyphenyllactic acid (4-OH-PLA), phenyllactic acid (PLA), and indole-3-lactic acid (ILA), which are produced by *Lactobacillus* and *Bifidobacterium*, have been reported as the ligands of HCA_3_ but not HCA_2_ [3,9].

*Bifidobacterium* is one of the major components of the human gut microbiota and is of substantial importance because of its purported health-promoting effects in humans across their lifespan [10]. *Bifidobacterium* species that are naturally present in the human gut have been categorized as human-residential bifidobacteria (HRB), whereas other species, which are the natural inhabitants of animals or the environment, are non-HRB [10,11,12]. HRB is most abundant in the human gut during infancy and decreases with age [12]. Bifidobacteria produce several aryl-LA metabolites, such as ILA, which is a metabolite of tryptophan [13]. It has been reported that the ILA produced by HRB is effective in improving necrotizing enterocolitis through its anti-inflammatory effect [14,15,16,17] and may function in modulating the immune balance early in the life of breastfed infants [18].

In this study, we examined HCA_3_ ligands in the culture supernatants (CSs) of *Bifidobacterium* strains. The HCA_3_ ligand activity of the enantiomers and chemical intermediates of the metabolites was also examined.

## 2. Materials and Methods

### 2.1. Chemicals

DL-4-OH-PLA, L-PLA, D-PLA, phenylpyruvic acid (PpyA), 4-hydroxyphenylpyruvic acid (4-OH-PpyA), and L-leucic acid were purchased from Tokyo Chemical Industry Co., Ltd. (Tokyo, Japan). D-Leucic acid (LeuA) was purchased from the ChemSpace.com database (Riga, Latvia). DL-2-hydroxycaproic acid (2-OH-HA), DL-ILA, 3-methyl-2-oxindol, indole-3-pyruvic acid (IpyA), l-kynurenine, and *p*-hydroxyphenylpyruvic acid were purchased from Merck KGaA (Darmstadt, Germany). The optical isomers of ILA, 1st-ILA, and 2nd-ILA, were prepared by the Daicel Corporation (Osaka, Japan). Acetonitrile (high-performance liquid chromatography [HPLC] grade) was purchased from Kanto Chemical Co., Ltd. (Tokyo, Japan). Ammonium acetate, which is suitable for mass spectrometry, was purchased from Merck KGaA. Unless otherwise stated, all chemical reagents used were of analytical grade.

### 2.2. Bacterial Strains

*Bifidobacterium* strains were purchased from the American Type Culture Collection (ATCC, Manassas, VA, USA), Japan Collection of Microorganisms (JCM, Wako, Japan), and Deutsche Sammlung von Mikroorganismen und Zellkulturen (DSM, Braunschweig, Germany) or obtained from the Morinaga Culture Collection (Morinaga Milk Industry Co., Ltd., Zama, Tokyo, Japan). Some strains were deposited in the culture collection of the National Institute of Technology and Evaluation (NITE, Tokyo, Japan). Strains were cultured under anaerobic conditions in Man, Rogosa, and Sharpe (MRS) broth (BD Biosciences, Franklin Lakes, NJ, USA) supplemented with 0.05% L-cysteine (Kanto Chemical Co., Ltd.) (MRS-C) using Anaero Pack (Mitsubishi Gas Chemical, Tokyo, Japan).

### 2.3. CSs

CSs were collected as described previously [19]. Briefly, *Bifidobacterium* strains were cultured at 37 °C for 16 h under anaerobic conditions in the MRS-C. The CSs were obtained by centrifuging the culture suspensions, followed by filtration (pore size 0.22 μm; Millipore, Billerica, MA, USA), and then stored at −80 °C until use. All CSs were individually prepared three times and processed for analysis.

### 2.4. Quantification of Metabolite Concentrations

The concentrations of the metabolites in the CSs and fractionated samples were analyzed using liquid chromatography–tandem mass spectrometry (LC-MS/MS; Vanquish HPLC connected with TSQ-FORTIS, Thermo Fisher Scientific, Waltham, MA, USA). Chromatographic separation was performed using an XBridge^®^ C18 column (Waters Corporation, Milford, MA, USA) (4.6 × 150 mm, 3.5 μm). Mobile phase A (containing 1 g/L ammonium acetate in water) and mobile phase B (containing 1 g/L ammonium formate and 0.1% formic acid in methanol) were applied at a flow rate of 0.2 mL/min. Gradient elution was performed between 2% and 40% of phase B. Quantification was performed by comparing metabolite peak areas with those of the corresponding synthetic compound standards and internal standard (3-methyl-2-oxindole). The LC-MS/MS spectrum (product ion data) of the precursor ion was evaluated to determine the final content of each metabolite (Table S1).

### 2.5. Analysis of the Optical Isomer of Aryl-LAs Using Chiral HPLC

**Figure 1:**
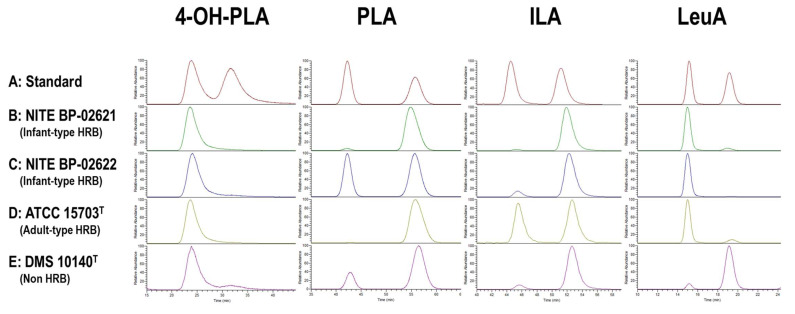
Chiral chromatography of aryl-lactic acids (aryl-LAs) and leucic acid (LeuA). The earlier retention time peak is labeled as “1st.” (**A**) Authentic standards for DL-4-OH-PLA, PLA, and ILA. (**B**–**E**) CS samples of *B. longum* subsp. *longum* (NITE BP-02621), *B. breve* (NITE BP-02622), *B. adolescentis* (ATCC15703^T^), and *B. animalis* subsp. *lactis* (DSM10140^T^).

The chiral columns (Daicel Corporation) and the conditions used to analyze each aryl-LA are listed in Table S2. Before chiral column analysis, the CSs were prepared as follows: (i) 1 mL of the CSs was mixed with 9 mL of methanol (Wako, Osaka, Japan), (ii) aggregates were removed via centrifugation (10,000× *g* for 5 min) (TOMY MX-307, Tomy Seiko Co., Ltd., Tokyo, Japan); (iii) the supernatants were evaporated (miVac Quattro LV, Genevac Ltd., Ipswich, UK), and the dried samples were reconstituted in 1.1 mL ultra-pure water (Wako) and immediately filtered (pore size 0.22 μm; Millipore); and (iv) the samples were applied (1.0 mL) to the HPLC column with a diode array using preparative reversed-phase chromatography on a Waters e2695 with a PDA detector (2998) and Waters Fraction Collector III and XBridge^®^ C18 OBD Prep column (10 × 250 mm, 5 μm) (Waters Corporation). Mobile phases A and B, as described above, were applied at 3.0 mL/min. Gradient elution was performed between 2% and 20% phase B. The retention time of the aryl-LA elution was confirmed beforehand, and samples were collected from 5.5–7.5 min (4-OH-PLA), 11.5–13.0 min (PLA), and 13.5–15.0 min (ILA) (Figure S2). Each sample collected was evaporated, and the dried samples were reconstituted in 0.2 mL of ultra-pure water (Wako) and filtered (pore size 0.22 μm; Millipore), followed by storage at −80 °C until use. Finally, the prepared samples were analyzed using a chiral column (10 μL injection), as described in Table S2. The chiral column was connected to an LC-MS/MS system (Vanquish HPLC and TSQ-FORTIS). The LC-MS/MS spectrum (product ion data) of the precursor ion was evaluated to represent the elution peaks in the ion chromatograms (Table S2). The typical elution patterns of the aryl-LA standards and samples are shown in Figure 1.

### 2.6. Preparative LC of MRS and CSs

**Figure 2:**
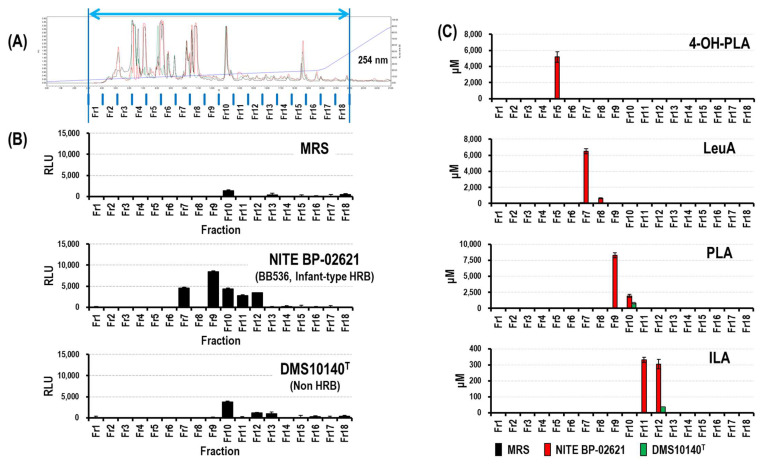
Fractionation of culture supernatants of *Bifidobacterium* strains. (**A**) HPLC, HPLC-DAD chromatograms (detector λ254 nm) of CSs and MRS. CS of *B. longum* subsp. *longum* (NITE BP-02621, infant-type HRB) (red line) and CS of *B. animalis* subsp. *lactis* (DSM10140^T^, non-HRB) (green line) and MRS (black line). The fractions were collected every 1 min (from 3–22 min) and numbered from fractions 1–18. (**B**) HCA_3_ activity of the 18 fractions of MRS and CSs of NITE BP-02621 and DSM10140^T^. The values shown are the mean values (RLU) of triplicates ± SD. RLU, relative light units that were subtracted from the PBS baseline. (**C**) Concentration of 4-OH-PLA, LeuA, PLA, and ILA in 18 fractions. Fractions MRS (black); NITE BP-02621 (red); and DSM10140^T^ (green). The values shown are the mean values (μM) of triplicates ± SD.

Each sample (MRS and CS) was fractionated using reversed-phase HPLC to analyze the optical isomers of the aryl-LAs. Before applying the samples, 45 mL of methanol (Wako) was mixed with 5 mL of each sample. The aggregates were removed via centrifugation (10,000× *g*, 5 min). The supernatants were evaporated, and the dried samples were reconstituted in 2 mL ultrapure water. The prepared samples were filtered (pore size 0.22 μm; Millipore) immediately before fractionation. One milliliter of the prepared samples was applied to an XBridge^®^ C18 OBD Prep column (10 × 250 mm, 5 μm). Mobile phases A and B, as described above, were applied at a flow rate of 3.0 mL/min to equilibrate the column. The gradient elution was started at 2% phase B. Between 2% and 20% of phase B, the metabolites were eluted. The fractions were collected every 1 min from 3–21 min, as shown in Figure 2A. Eighteen fractions of each sample were evaporated, and the dried samples were reconstituted in 0.3 mL of Dulbecco’s phosphate-buffered saline (Nacalai Tesque, Kyoto, Japan), filtered (pore size 0.22 μm; Millipore), and stored at −80 °C.

### 2.7. HCA_3_ Ligand Assay

The ligand activity for HCA_3_ was assayed using the PathHunter CHO-K1 GPR109B β-arrestin cell line, which was purchased from Eurofins DiscoverX Products, LLC (Fremont, CA, USA). The cells were engineered to co-express ProLink™-tagged G-protein-coupled receptors and the enzyme acceptor-tagged β-arrestin. The assay was performed according to the manufacturer’s instructions. Briefly, culture was performed in Corning^®^ (New York, NY, USA) 96-well plates (tissue culture-treated, half-area black plate with clear flat bottom and lid) (Corning). HCA_3_ ligand activity was detected using the PathHunter Detection Kit by measuring the generated chemiluminescent signal using an SH-9000 microplate reader (Colona Electric, Inc., Tokyo, Japan). The chemiluminescent signal is expressed as relative light units (RLU).

### 2.8. Aryl Hydrocarbon Receptor (AhR) Ligand Assay

The ligand activity for the AhR was assayed using HT29-Lucia™ AhR Cells, purchased from InvivoGen (San Diego, CA, USA). The cell lines were engineered to study AhR induction by monitoring the activity of Lucia luciferase reporter protein. QUANTI-Luc™ was used to detect the secreted luciferase (InvivoGen) according to the supplier’s instructions. Briefly, the assay was performed using Nunc™ MicroWell™ 96-well plates (#167008, Thermo Fisher Scientific). Chemiluminescent signals were measured using Nunc™ FluoroNunc™/LumiNunc™ 96-well plates (#437796, Thermo Fisher Scientific) and an SH-9000 microplate reader (Colona Electric). The chemiluminescent signal is expressed as RLU.

### 2.9. Construction of Insertional Mutant

**Figure 3:**
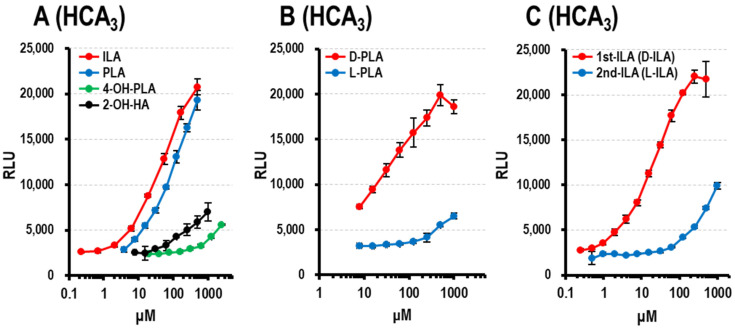

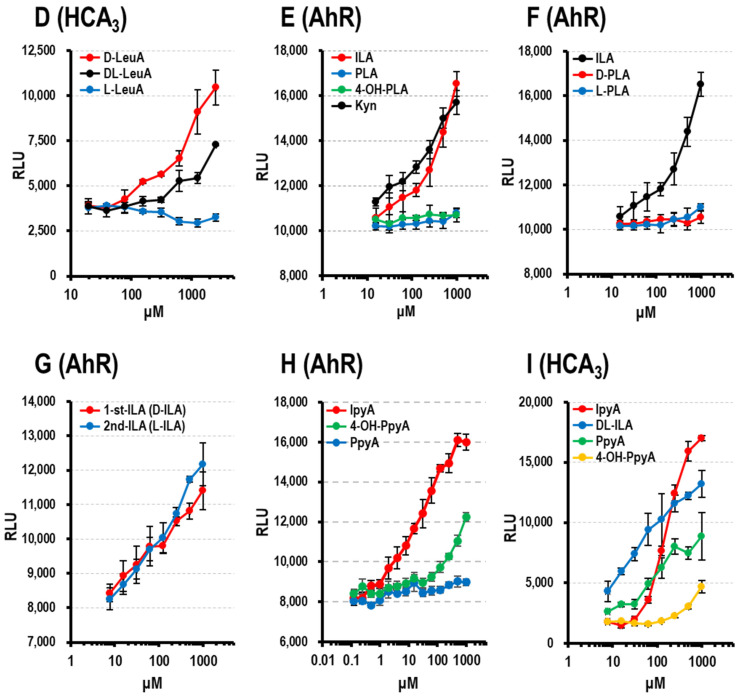
HCA_3_ ligand activity and AhR ligand activity. (**A**) Effect of increasing concentrations of aryl-LAs (racemic mixture) and DL-2-hydroxycaproic acid (2-OH-HA) on HCA_3_ activation. ILA (red circles), PLA (blue circles), 4-OH-PLA (green circles), and 2-OH-HA (black circles). (**B**) Effect of increasing concentrations of (D)-PLA (1st-PLA, red circles) and (L)-PLA (2nd-PLA, blue circles) on HCA_3_ activation. (**C**) Effect of increasing concentrations of 1st-ILA (D-ILA) (red circles) and 2nd-ILA (L-ILA) (blue circles) on HCA_3_ activation. (**D**) Effect of increasing concentrations of D-LeuA (red circles), L-LeuA (blue circles), and DL-LeuA (black circles) on HCA_3_ activation. (**E**) Effect of increasing concentrations of aryl-LAs (racemic mixture) and Kyn (L-kynurenine) on HCA_3_ activation. ILA (red circles), PLA (blue circles), 4-OH-PLA (green circles), and Kyn (black circles). (**F**) Effect of increasing concentrations of ILA (black circles), (D)-PLA (1st-PLA, red circles), (L)-PLA (2nd-PLA, blue circles), and ILA (racemic mixture, black circles) on HCA_3_ activation. (**G**) Effect of increasing concentrations of 1st-ILA (D-ILA) (red circles) and 2nd-ILA (L-ILA) (blue circles) on AhR activation. (**H**) Effect of increasing concentrations of IpyA (red circles), PpyA (blue circles), and 4-OH-PpyA (green circles) on AhR activation. (**I**) Effect of increasing concentrations of IpyA (red circles), PpyA (blue circles), 4-OH-PpyA (green circles), and DL-ILAs (black circles) on HCA_3_ activation. RLU, relative light units. The data are shown as the mean values of triplicates ± SD.

Figure 3C shows that the HCA_3_ ligand activity of the ILA enantiomers was different. However, we could not determine which peak was L-ILA in the chiral HPLC analysis of CSs, because the synthetic standards of L-ILA and D-ILA were not commercially available. In contrast, type 4 L-LDH has been reported to be involved in the production of ILA by infant-type HRB [9]. We constructed two insertional mutants by disrupting type 4 L-LDH and D-2-hydroxyacid dehydrogenase (D-2-OH-A-DHH). The involvement of D-2-OH-A-DHH has not been reported in the production of ILA; however, the D-2-OH-A-DHH subfamily has been reported to catalyze the reduction of a broad range of 2-ketocarboxylic acids [20]. Briefly, we expected that D-2-OH-A-DHH would be involved in the production of D-ILA. The insertional mutation of genes presumed to be involved in aryl-LAs production was carried out by plasmid-mediated single crossover recombination, as described previously [21]. The plasmid used for disruption was constructed using the In-Fusion cloning kit (Clontech Laboratories, Inc., Mountain View, CA, USA). *Escherichia coli* DH5α was used as the host. The internal regions of BL105A_0985 (type 4 *ldh*) and BL105A_1367 (D-2-OH-A-DHH) genes were amplified using PCR from the genomic DNA of *B. longum* subsp. *longum* 105-A and ligated with SacI -andNcoI-digested pKKT427 with fragments containing pUC ori and the spectinomycin resistance gene [22]. The resulting suicide plasmids were independently introduced into *B. longum* subsp. *longum* 105-A by electroporation, and respective insertional gene mutants were subsequently selected on Gifu anaerobic medium agar plates (Nissui Pharmaceutical Co., Ltd., Tokyo, Japan) containing 30 μg mL^−1^ spectinomycin. The insertional gene disruption was verified using PCR and a primer pair designed to anneal outside of the gene. The primer pairs used are listed in Table S3.

## 3. Results

### 3.1. HCA_3_ Ligand Activity and Aryl-LAs Concentration of CSs of Bifidobacterium Strains

Two CSs of *Bifidobacterium* strains, *B. longum* subsp. *longum* NITE BP-02621 (infant-type HRB) and *B. animalis* subsp. *lactis* DSM10140^T^ (non-HRB), and an MRS control were prepared and fractionated using HPLC. As shown in Figure 2A, clear elution patterns were observed from 3–22 min (retention time); therefore, we collected aryl-LA fractions during this period (18 fractions), as shown in Figure 2A. Although there were no distinct peaks among the three samples (Figure 2A), we observed differences in activity between the two strains (Figure 2B). Strong HCA_3_ ligand activity was detected in the fractions of NITE BP-02621-CS (fractions 7, 9, 10, 11, and 12); however, relatively weak HCA_3_ ligand activity was observed in the fractions of DSM10140^T^-CS and trace HCA_3_ activity was detected in the fractions of control MRS.

The content of the HCA_3_ ligand (aryl-LAs) in the 18 fractions was examined using LC-MS/MS analysis (Figure 2C). Aryl-LAs were not detected in the MRS fractions. In contrast, a relatively high content of aryl-LAs was detected in NITE BP-02621-CS. The activity of fractions 9 and 10 may be related to the presence of PLA. The activity of fractions 11 and 12 may be related to the presence of ILA. Although fraction 5 contained 4-OH-PLA, HCA_3_ ligand activity was not observed, whereas this activity was observed in fraction 7, but no aryl-LAs were detected.

#### 3.1.1. Identification of Novel HCA_3_ Ligand in CSs of *Bifidobacterium* Strains (Fraction 7)

To explain the HCA_3_ ligand activity of fraction 7, the active compound in this fraction was purified using a combination of three different types of chromatographic techniques (phenyl-hexyl, Scherzo C18, and C18 column). Based on the molecular weight of the purified compound, two candidates were selected: 2-hydroxycaproic acid (2-OH-HA) and leucine acid (LeuA; 2-hydroxy-4-methylvaleric acid). 2-OH-HA activates HCA_3_ [7]. However, a comparison of the characteristic product ions of fraction 7 and 2-OH-HA ruled out the presence of 2-OH-HA and identified the compound as LeuA (Figure S1). The concentrations of LeuA in each of the 18 fractions were examined using LC-MS/MS analysis (Figure 2C). The concentration of LeuA in fraction 7 of NITE BP-02621-CS was higher than that of DSM10140^T^S (Figure 2C), which agrees with the HCA_3_ ligand activity of each fraction 7 (Figure 2B).

#### 3.1.2. Production of Aryl-LAs and LeuA by *Bifidobacterium* Strains

In a previous report, *Bifidobacterium* strains isolated from human infants (infant-type HRB) produced higher levels of aryl-LAs than *Bifidobacterium* strains isolated from non-human (non-HRB) [9]. We examined the production of aryl-LAs and LeuA in the CS of 19 *Bifidobacterium* strains (Table 1). Infant-type HRB produced significantly higher levels of 4-OH-PLA and ILA than adult-type HRB and non-HRB; however, the levels of PLA and LeuA did not differ between HRB and non-HRB.

#### 3.1.3. Optical Activity of Aryl-LAs and LeuA in CSs of *Bifidobacterium* Strains

The optical activity of aryl-LAs and LeuA produced by *Bifidobacterium* strains in CSs was examined using chiral columns. The elution pattern of the authentic standard compounds (mixture of enantiomers) showed two chromatographic peaks (Figure 2). To differentiate these peaks, we designated the earlier chromatographic peak as “1st” and the latter as “2nd.” Chromatography of five strains, including infant-type HRB, adult-type HRB, and non-HRB, showed that aryl-LAs and LeuA in the CSs were mixtures of enantiomers (Figure 2). The relative percentage of the 1st-peak (area under the curve) of the CSs of the 19 strains was calculated based on the total area under the curve (Table 2). The 1st-peak of 4-OH-PLA (1st-4-OH-PLA) was the highest among those of the examined 19 strains (over 85%), except for that of *B. dentium* (39.1 ± 7.2%). In contrast, the distribution of the 1st peak of PLA and ILA was strain dependent. Based on the total concentration of aryl-LAs in the CSs (Table 1) and their ratio of the 1st-peak (Table 2), the CS of *B. breve* strain LMG 23729 contained the highest concentration of 1st-PLA (14.2 ± 0.8 μM) and 1st-ILA (1.31 ± 0.19 μM) among the CS of 19 strains.

#### 3.1.4. HCA_3_ Ligand Activity of Aryl-LAs and Enantiomers of PLA, ILA, and LeuA

As has been previously reported [8], we confirmed the HCA_3_ ligand activity of aryl-LAs (Figure 3A) and 2-OH-HA. ILA and PLA showed stronger activities than 4-OH-PLA. D-PLA (1st-PLA) showed stronger HCA_3_ ligand activity than L-PLA (2nd-PLA) (Figure 3B), which is consistent with a previous report [3]. As shown in Figure 3C, the HCA_3_ ligand activity of 1st-ILA was stronger than that of the 2nd-ILA. We examined the HCA_3_ ligand activity of synthetic LeuA enantiomers and their mixtures (Figure 3D). The HCA_3_ ligand activity of D-LeuA was stronger than that of L-LeuA and DL-LeuA.

#### 3.1.5. AhR Ligand Activity of Aryl-LAs

ILA has been reported to be a ligand of AhR [15,17,23,24]. We confirmed that ILA activated AhR (Figure 3E) to a level similar to that of Kyn, which has been reported as an endogenous ligand of AhR [25]. In contrast, 4-OH-PLA and PLA did not show obvious AhR activity (Figure 3E). Neither of the two PLA enantiomers showed AhR activity (Figure 3F). Similarly, no difference was observed in the AhR ligand activity of the two enantiomers of ILA (Figure 3G).

#### 3.1.6. AhR and HCA_3_ Ligand Activity of Aryl-pyr-As

Aryl-pyr-As are the intermediate metabolites of aryl-LAs [13]. Aryl-pyr-As have been reported as endogenous metabolite mediators in the host, such as AhR ligands [26,27]. Using our assay system, we confirmed the ligand activity of IpyA against AhR (Figure 3H). In addition, as shown in Figure 3I, aryl-pyr-As stimulated HCA_3_. In particular, the HCA_3_ ligand activity of IpyA was stronger than that of ILA.

## 4. Discussion

The compounds that activate HCA_3_ in the CSs of *Bifidobacterium* were investigated. As shown in Figure 2B, the CS fractions of *B. longum* subsp. *longum* NITE BP-02621 (infant-type HRB) showed more explicit activity than the CS fractions of *B. animalis* subsp. *lactis* DSM10140^T^ (non-HRB). Since the HCA_3_ ligand activity of aryl-LAs was previously described [3,9], the difference in their activity (Figure 2B) was well reflected by the aryl-LA levels in the fractions (Figure 2C) and CSs (Table 1). Although 4-OH-PLA was detected in CS fraction 5 (Figure 2C, Table 1), HCA_3_ ligand activity was not detected in this fraction (Figure 2B). This may be because the levels of 4-OH-PLA in the fractions were too low to show activity in our HCA_3_ ligand assay (Figure 3A). Although HCA_3_ ligand activity was observed in fraction 7 of NITE BP-02621-CS, aryl-LAs were not detected. Further purification and LC mass spectrometry analysis led to the identification of LeuA as an active component (Figure S1). Furthermore, the HCA_3_ ligand activity of D-LeuA was stronger than that of L-LeuA (Figure 3D). To the best of our knowledge, the HCA_3_ ligand activity of LeuA has not yet been reported. LeuA is produced by *Bifidobacterium* strains as a metabolite of L-leucine in the formation of the flavor compound 3-methylbutanal [28]. The broad antifungal activity of LeuA has been demonstrated against *Candida* and *Aspergillus* [29]. Increased LeuA levels were also reported in the saliva of Japanese patients with oral squamous cell carcinoma and the fecal metabolites of subjects with an elderly type gut microbiota [30,31]. However, their optical activity remains unclear. Further examination of the involvement of LeuA in host homeostasis is required.

The constitutional formula of aryl-LAs indicates that they have two structural enantiomers. Although the production of aryl-LAs from *Bifidobacterium* strains has been reported, information regarding their optical activity is not clear. The CSs of *Bifidobacterium* strains were examined, and we found that the optical activity of aryl-LAs and LeuA in the CSs was a mixture of enantiomers (Figure 2, Table 2). As mentioned later, since the optical activity influenced the HCA_3_ ligand activity, the production of a mixture of enantiomers should be considered to evaluate the relationship between the host and their commensal bifidobacteria. We could not show that the 1st-peak of ILA was D-ILA because their synthetic standards were not commercially available. The gut bacterial metabolic pathway of aromatic amino acids (aromatic AAs) to aromatic lactic acids (aryl-LAs) has been explained in two steps [13]. First, the deamination of aromatic AA by aminotransferases. This step generates arylpyruvate acids (aryl pyr-As) from aromatic AAs. Second, lactate dehydrogenases (LDHs) convert aryl pyr-As to aryl-LAs. We suppose that the productive distribution of the aryl-LA enantiomers depends on the active properties of LDHs and similar enzymes, such as D-2-OH-A-DHH [20]. In the preliminary examination, the type 4 L-LDH gene of *B. longum* subsp. *longum* 105-A was disrupted, and the ILA in the CS was examined. As shown in Figure S3, the 2nd-peak of their (Δ*ldh4*) ILA decreased in size. Since the type 4 L-LDH gene was reported to be involved in the production of ILA, we supposed that the 2nd-peak of ILA is L-ILA, and consequently the 1st-ILA is D-ILA; however, future X-ray structure analysis is needed for confirmation. We also tried to disrupt the D-2-OH-A-DHH gene. Since the subfamily of D-2-OH-A-DHH has been reported to catalyze the reduction of a broad range of 2-ketocarboxylic acids [20], we expected that D-2-OH-A-DHH may be involved in the production of D-ILA. As shown in Figure S3, the 1st-peak of their (ΔD-2-OH-A-DHH) ILA decreased in size, which indicates that the 1st-ILA is D-ILA. However, since the relative percentage of the 1st-peak of the wild type was only 2%, further examination of the involvement of D-2-OH-A-DHH is needed to confirm these speculations. In addition, there should be many enzymes involved in the production of D-ILA [32], and further examination of the production of D-aryl-LAs is needed.

The HCA_3_ ligand activity of aryl-LAs is shown in Figure 3A. As reported previously, the HCA_3_ ligand activity of D-PLA is stronger than that of L-PLA [3]. The HCA_3_ ligand activity of ILA (racemic mixture) was similar to that of PLA (Figure 3A). However, the difference in the activity of the two ILA enantiomers remains unclear. We found that the HCA_3_ ligand activity of 1st-ILA (D-ILA) was stronger than that of 2nd-ILA (L-ILA) (Figure 3C); however, there was no marked difference in the activity against AhR between the two ILA enantiomers [23]. To the best of our knowledge, this is the first report on the difference in HCA_3_ ligand activity between ILA enantiomers. Further studies are needed to evaluate the relationship between the metabolites of microbiota and their host receptors.

## 5. Conclusions

In conclusion, *Bifidobacterium* strains were shown to produce LeuA in addition to aryl-LAs as HCA_3_ ligands. The aryl-LAs and LeuA produced by *Bifidobacterium* are mixtures of enantiomers. The HCA_3_ ligand activity of PLA, ILA, and LeuA depended on their optical structures, with stronger activities in the D-isomers. We showed that there was no difference in AhR activity between the two ILA enantiomers. However, our results were obtained under limited in vitro conditions and need to be verified in the human body.

## Figures and Tables

**Table 1:** Production of HCA_3_ ligands in culture supernatants of *Bifidobacterium* strains.

				Concentration (μM)			
Species	Isolation Source	Strain	OD_600_	4-OH-PLA	PLA	ILA	LeuA
Infant-type HRB							
*B. bifidum*	Infant feces	ATCC29521^T^	0.78 ± 0.03	14.5 ± 1.3	492.9 ± 127.5	19.0 ± 5.1	131.6 ± 8.2
	Infant feces	NITE BP-02429	0.84 ± 0.05	6.8 ± 0.6	184.5 ± 25.5	7.4 ± 1.9	26.9 ± 6.2
	Infant feces	NITE BP-02431	1.10 ± 0.02	17.6 ± 1.8	248.3 ± 57.5	13.8 ± 3.6	60.8 ± 12.1
*B. breve*	Intestine of infant	ATCC15700^T^	1.24 ± 0.03	4.2 ± 1.3	23.0 ± 3.6	10.0 ± 2.1	99.3 ± 6.6
	Infant feces	FERM BP-11175 (MCC 1274)	1.22 ± 0.02	12.6 ± 2.5	26.1 ± 4.2	9.5 ± 2.3	48.8 ± 2.4
	Infant feces	NITE BP-02622 (M-16V)	1.33 ± 0.01	8.1 ± 2.0	33.3 ± 7.3	10.6 ± 2.7	122.6 ± 4.7
*B. longum* subsp. *infantis*	Intestine of infant	ATCC15697^T^	1.35 ± 0.02	16.2 ± 3.7	63.3 ± 11.3	6.9 ± 2.0	44.1 ± 1.1
	Intestine of infant	NITE BP-02623 (M-63)	1.34 ± 0.02	12.9 ± 2.7	72.3 ± 13.6	6.6 ± 1.9	63.2 ± 2.3
*B. longum* subsp. *longum*	Intestine of adult	ATCC15707^T^	1.14 ± 0.01	8.9 ± 2.4	123.0 ± 20.8	23.6 ± 5.0	181.8 ± 18.1
	Infant feces	NITE BP-02621 (BB536)	1.24 ± 0.03	23.6 ± 2.9	152.2 ± 11.4	10.2 ± 1.9	230.1 ± 38.7
Average of infant-type HRB			1.16 ± 0.2	12.5 ± 5.7	141.9 ± 144.2	11.7 ± 5.6	78.2 ± 65.9
Adult-type HRB							
*B. adolescentis*	Intestine of adult	ATCC15703^T^	1.20 ± 0.01	2.2 ± 1.2	18.2 ± 3.9	0.2 ± 0.1	46.1 ± 3.2
*B. angulatum*	Feces, human	ATCC27535^T^	1.18 ± 0.03	10.7 ± 1.0	72.4 ± 10.3	4.6 ± 1.7	255.6 ± 34.2
*B. dentium*	Dental caries	DSM20436^T^	1.14 ± 0.05	0.7 ± 0.7	9.9 ± 2.0	0.9 ± 0.3	112.7 ± 12.2
*B. pseudocatenulatum*	Feces, human	ATCC27919^T^	1.19 ± 0.04	0.6 ± 0.9	14.8 ± 4.2	0.8 ± 0.2	136.4 ± 14.9
Average of adult-type HRB			1.18 ± 0.02	3.6 ± 4.8 *	28.8 ± 29.2	1.6 ± 2.0 **	137.7 ± 87.4
Non-HRB							
*B. animalis* subsp.*lactis*	yoghurt	DSM10140^T^	1.03 ± 0.00	2.6 ± 1.0	16.6 ± 3.2	0.9 ± 0.1	60.1 ± 12.9
*B. animalis* subsp. *animalis*	Rat feces	ATCC25527^T^	1.05 ± 0.02	0.7 ± 0.5	18.6 ± 4.2	0.7 ± 0.2	103.0 ± 10.4
*B. pseudolongum* subsp. *globosum*	Rumen	JCM5820^T^	0.85 ± 0.02	1.5 ± 0.7	8.9 ± 2.8	1.0 ± 0.8	46.8 ± 2.3
*B. pseudolongum* subsp. *pseudolongum*	Swine feces	ATCC25526^T^	0.95 ± 0.01	4.6 ± 0.9	35.4 ± 3.9	1.3 ± 0.2	216.9 ± 36.8
*B. thermophilum*	Swine feces	ATCC 25525^T^	1.25 ± 0.03	0.6 ± 0.3	23.3 ± 3.9	1.3 ± 0.3	170.9 ± 29.3
Average of non-HRB			1.03 ± 0.15	1.98 ± 1.69 **	20.53 ± 9.78	1.04 ± 0.27 ***	119.55 ± 72.85

Concentrations and culture growth (OD_600_) are expressed as the mean ± SD. * *p* < 0.05, ** *p* < 0.01, *** *p* < 0.001, significant difference vs. infant-type HRB.

**Table 2:** Relative percentage of the 1st peak area of aryl-lactic acids (aryl-LAs) in the culture supernatants of *Bifidobacterium* strains.

		1st %			
Species	Strain	4-OH-PLA	PLA	ILA	LeuA
*B. bifidum*	ATCC29521^T^	96.9 ± 0.0	0.7 ± 0.1	0.3 ± 0.2	41.5 ± 2.4
	NITE BP-02429	96.0 ± 1.5	0.2 ± 0.0	0.3 ± 0.1	32.4 ± 2.0
	NITE BP-02431	96.8 ± 0.6	0.3 ± 0.0	0.3 ± 0.0	31.8 ± 2.1
*B. breve*	ATCC15700^T^	95.9 ± 2.4	44.3 ± 2.3	8.4 ± 0.9	97.8 ± 0.2
	FERM BP-11175 (MCC 1274)	96.8 ± 0.1	13.8 ± 0.6	3.4 ± 0.2	94.5 ± 0.2
	NITE BP-02622 (M-16V)	95.6 ± 1.5	39.2 ± 3.5	10.8 ± 1.0	98.1 ± 0.4
*B. longum* subsp. *infantis*	ATCC15697^T^	96.4 ± 0.3	1.9 ± 0.1	0.7 ± 0.0	84.7 ± 1.3
	NITE BP-02623 (M-63)	97.7 ± 1.7	2.9 ± 0.1	0.8 ± 0.0	88.1 ± 1.2
*B. longum* subsp. *longum*	ATCC15707^T^	98.3 ± 2.3	2.9 ± 0.1	2.1 ± 0.1	92.8 ± 0.2
	NITE BP-02621 (BB536)	95.8 ± 0.1	2.8 ± 0.1	2.7 ± 0.2	92.2 ± 0.7
*B. adolescentis*	ATCC15703^T^	96.2 ± 4.0	4.3 ± 0.3	87.6 ± 13.8	89.6 ± 0.9
*B. angulatum*	ATCC27535^T^	96.9 ± 0.2	7.1 ± 0.5	1.1 ± 0.2	95.2 ± 0.5
*B. dentium*	DSM20436^T^	39.1 ± 7.2	77.5 ± 3.5	99.9 ± 0.0	96.2 ± 0.2
*B. pseudocatenulatum*	ATCC27919^T^	92.8 ± 8.6	21.3 ± 2.2	63.4 ± 2.5	49.6 ± 3.5
*B. animalis* subsp. *lactis*	DSM10140^T^	98.2 ± 3.4	4.3 ± 0.3	8.7 ± 1.3	14.1 ± 2.5
*B. animalis* subsp. *animalis*	ATCC25527^T^	98.2 ± 2.8	5.1 ± 0.6	13.7 ± 1.1	7.7 ± 0.3
*B. pseudolongum* subsp. *globosum*	JCM5820^T^	95.0 ± 0.4	26.2 ± 3.0	68.2 ± 5.4	73.3 ± 1.5
*B. pseudolongum* subsp. *pseudolongum*	ATCC25526^T^	98.0 ± 3.2	1.3 ± 0.1	8.5 ± 0.2	12.5 ± 0.2
*B. thermophilum*	ATCC 25525^T^	87.8 ± 4.1	28.6 ± 3.0	81.0 ± 9.2	96.9 ± 0.1

Aryl-LA enantiomers were analyzed as described in the Materials and Methods section. The percentage of the earlier eluting peak (area under the curve), herein designated as “1st peak”, was calculated using the following formula: The area under the curve of the 1st peak/total area under curve × 100. Results are expressed as the mean ± SD.

## Data Availability

Data are available upon reasonable request from the corresponding author.

## Supplementary Materials

The following are available online at https://www.mdpi.com/article/10.3390/microorganisms9112397/s1, Figure S1: LC-MS/MS comparison of Fraction 7 and LeuA, and 2-OH-HA; Figure S2: Partial purification of Aryl-LAs in the CSs; Figure S3: Chiral chromatography of Aryl-Las, Table S1: List of metabolites and internal standard; Table S2: List of chiral columns and analysis conditions used in the study; Table S3: Primer pairs used for gene disruption and confirmation.
