# Supplementary material for: Production of Hydroxycarboxylic Acid Receptor 3 (HCA3) Ligands by Bifidobacterium
Source: Microorganisms. 2021 Nov 21;9(11):2397. doi: 10.3390/microorganisms9112397 (PMC8620054; doi:10.3390/microorganisms9112397)
Supplement: Supplementary file 1 [file microorganisms-09-02397-s001.zip › Figure S1-3.pptx]

## Slide 1
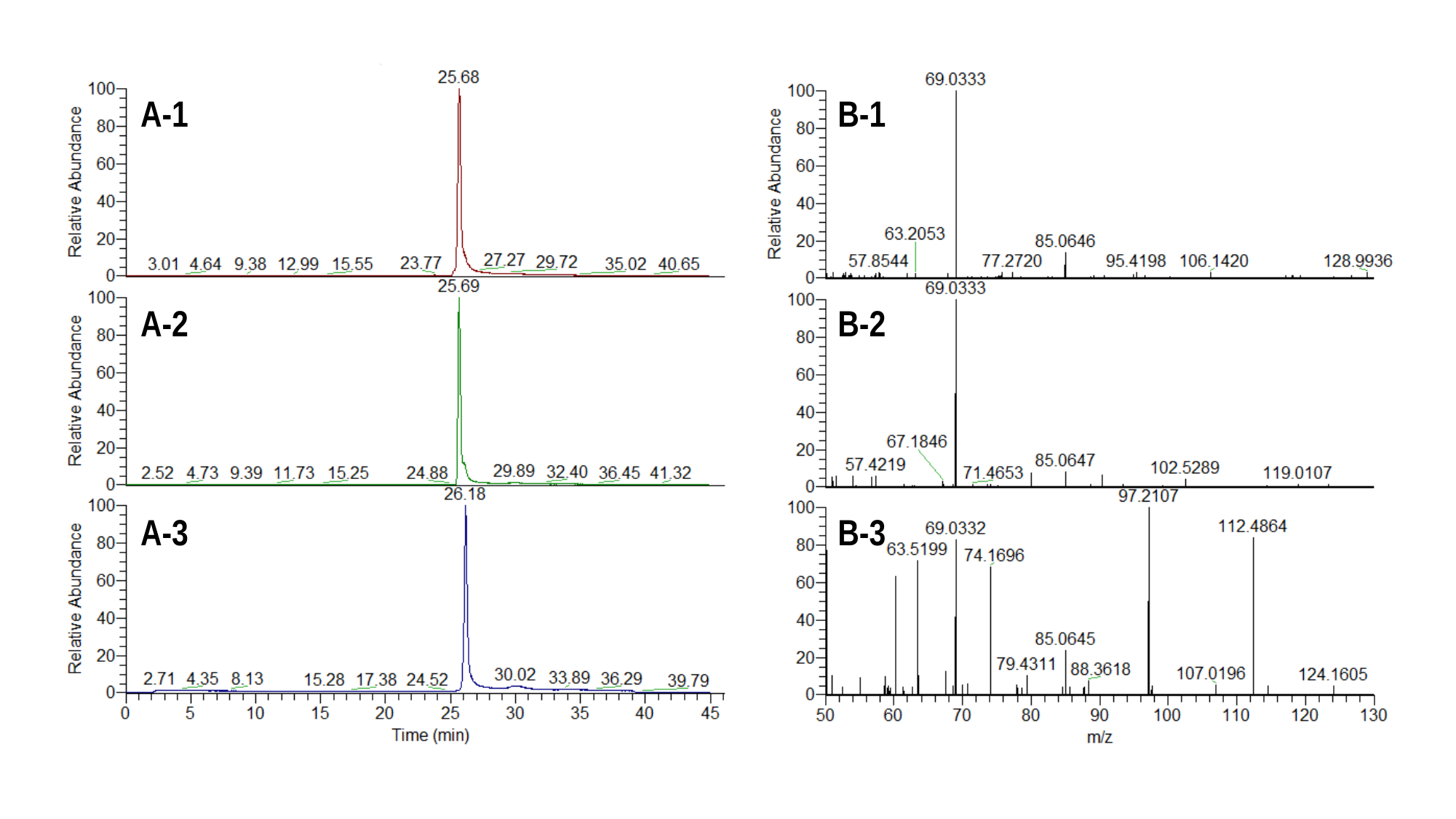

A-1
B-1
A-2
B-2
A-3
B-3

## Slide 2
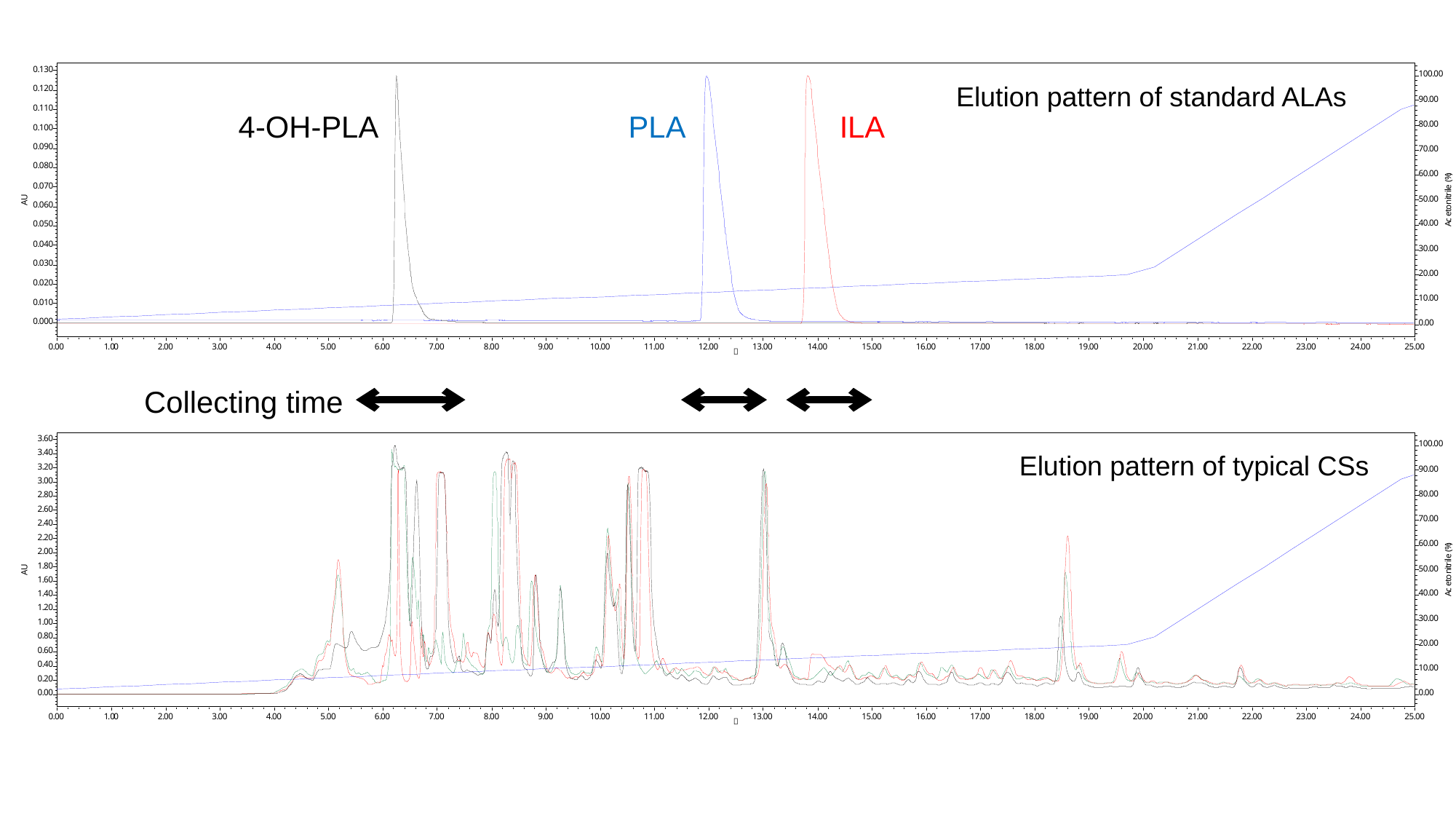

Elution pattern of standard ALAs
4-OH-PLA
PLA
ILA
Collecting time
Elution pattern of typical CSs

## Slide 3
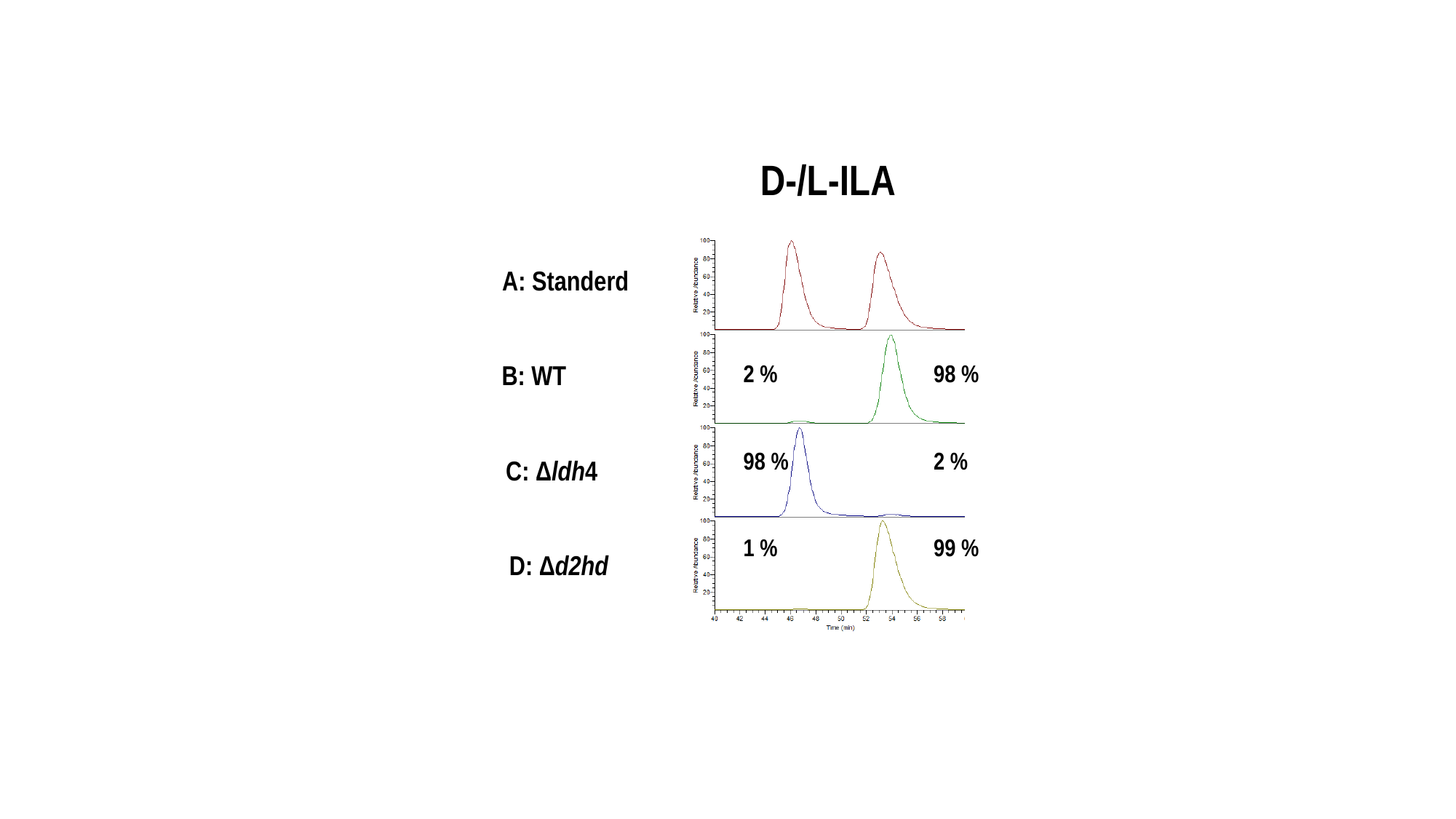

D-/L-ILA
A: Standerd
B: WT
C: Δldh4
D: Δd2hd
2 %
98 %
98 %
2 %
1 %
99 %
