# Supplementary material for: Production of Hydroxycarboxylic Acid Receptor 3 (HCA3) Ligands by Bifidobacterium
Source: Microorganisms. 2021 Nov 21;9(11):2397. doi: 10.3390/microorganisms9112397 (PMC8620054; doi:10.3390/microorganisms9112397)
Supplement: Supplementary file 1 [file microorganisms-09-02397-s001.zip › Tables.pdf]

Table S1. List of metabolites and internal standard

Name	Compounds Abbreviation	Polarity	Precursor (m/z)	Product (m/z)	Collision energy (V)	CAS registry number
4-hydroxyphenyllactic acid	4-OH-PLA	Positive	200.093	91.167	30.57	306-23-0
2-hydroxy-4-methylvaleric acid	LeuA	Negative	131.050	85.054	11.44	13748-90-8
3-phenyllactic acid	PLA	Positive	184.079	121.054	14.18	20312-36-1
indole-3-lactic acid	ILA	Positive	206.100	130.125	28.93	1821-52-9
3-methyl-2-oxindole	MO	Positive	148.071	133.054	20.21	1504-06-9

Table S2. List of chiral columns and analysis conditions used in the study.

Aryl-LA	Column	Mobile phase A H_2O, 0.2% FA	Mobile phase B Methanol	Flow rate (mL/min.)	Polarity	Precursor (m/z)	Product (m/z)	Collision energy (V)
4-OH-PLA	CHIRAKPAK IF-3	86%	14%	0.4	Negative	181.000	163.042	11.11
PLA	CHIRAKPAK IN-3	70%	30%	0.5	Negative	165.029	147.054	10.01
ILA	CHIRAKPAK IB N-3	70%	30%	0.5	Positive	206.093	130.125	30.06
LeuA	CHIRAKPAK IN-3	70%	30%	0.5	Negative	131.050	85.125	11.57

FA, formic acid.

Table S3. Primer pairs used for gene disruption and confirmation

Target gene of *B. longum* subsp. *longum* 105-A	Forward primer[a]	Reverse primer[a]
For gene disruption		
BL105A_0985	GCAGAGTACTGAGCTgcccgcgacctcgatgat	TTTGCTCAGAATTCGtggtggacacggaggcga
BL105A_1367	GCAGAGTACTGAGCTttgcagaccttcagcgccg	TTTGCTCAGAATTCGaggcgtcagccaaggcctcg
For confirmation of gene disruption		
BL105A_0985	cctcgacatgatgaccaagtt	ggttgatggtgtcgatgct
BL105A_1367	ggttgtcccttggtttggt	tttcggattgcccgataag

BL105A_0985 (type 4 *ldh*) and BL105A_1367 (D-2-hydroxyacid dehydrogenase) genes were amplified by PCR from the genomic DNA of B. longum subsp. longum 105-A and ligated with SacI and NcoI -digested pKKT427 [31].
